# The Effect of Methyl-β-cyclodextrin on Apoptosis, Proliferative Activity, and Oxidative Stress in Adipose-Derived Mesenchymal Stromal Cells of Horses Suffering from Metabolic Syndrome (EMS)

**DOI:** 10.3390/molecules23020287

**Published:** 2018-01-30

**Authors:** Joanna Szydlarska, Christine Weiss, Krzysztof Marycz

**Affiliations:** 1 Department of Experimental Biology and Electron Microscope Facility, The Faculty of Biology and Animal Science, Wroclaw University of Environmental and Life Sciences, 50-631 Wroclaw, Poland; joanna.szydlarska@gmail.com (J.S.); d.weiss@horsedoc.ch (C.W.); 2 Wroclaw Research Centre EIT+, 54-066 Wroclaw, Poland

**Keywords:** equine metabolic syndrome, mesenchymal stem cells, methyl-β-cyclodextrin, cyclodextrin, stem cells

## Abstract

Methyl-β-cyclodextrin (MβCD) is a cyclic oligosaccharide, commonly used as a pharmacological agent to deplete membrane cholesterol. In this study, we examined the effect of MβCD on adipose-derived mesenchymal stromal cells (ASCs) isolated form healthy horses (ASC_CTRL_) and from horses suffering from metabolic syndrome (ASC_EMS_). We investigated the changes in the mRNA levels of the glucose transporter 4 (GLUT4) and found that MβCD application may lead to a significant improvement in glucose transport in ASC_EMS_. We also showed that MβCD treatment affected GLUT4 upregulation in an insulin-independent manner via an NO-dependent signaling pathway. Furthermore, the analysis of superoxide dismutase activity (SOD) and reactive oxygen species (ROS) levels showed that MβCD treatment was associated with an increased antioxidant capacity in ASC_EMS_. Moreover, we indicated that methyl-β-cyclodextrin treatment did not cause a dysfunction of the endoplasmic reticulum and lysosomes. Thereby, we propose the possibility of improving the functionality of ASC_EMS_ by increasing their metabolic stability.

## 1. Introduction

Nowadays, equine metabolic syndrome (EMS) has become one of the most common metabolic disorders in horses and has been steadily growing in the recent years. The disturbance of the metabolic balance is most likely due to the an improper, high-calorie diet, rich in sugar and concentrates, combined with limited exercise [1]. The excessive overweight, increased regional adiposity, insulin resistance (IR), and laminitis are the typical signs of EMS [2], but a definitive understanding of the underlying mechanisms of the metabolic syndrome still remains an open problem. It was assumed that EMS is caused by both environmental and genetic factors [3,4], thus molecular and epigenetic changes might be essential factors in its development.

Mesenchymal stromal cells (MSCs) are a population of multipotent cells, characterized by a high proliferative and differentiation potential, a great capacity for self-renewal, and the ability to modulate the immune response by secreting cytokines, antiapoptotic factors, and growth factors via membrane-derived vesicle (MVs) shedding [5]. Therefore, MSCs are considered a promising therapeutic tool, as well as an important cell source for regenerative medicine applications [6,7]. Recent data indicates MSCs cytophysiological impairment in the course of metabolic diseases [8,9] such as obesity and metabolic syndromes. Our laboratory’s previous studies demonstrated that EMS is closely associated with mitochondrial dysfunction, increased oxidative stress, and, consequently, disturbances in the proliferative activity of mesenchymal stem cells [3,10].

In the last decade, many studies have shown that metabolic disorders, such as insulin resistance, are directly related to the increased synthesis and reduced absorption of cholesterol [11]. It was shown that endogenous cholesterol production is significantly increased in diseased cells [12], which results in the accumulation and finally overload of lipids in the interior part of cellular bodies. The most important role of cholesterol is to maintain the stability of the lipid bilayer and thereby the structure of the whole cell [13], but cholesterol is also a precursor of steroid hormones. There is evidence that fat deposition is associated with the presence of specific hormones [14,15] that are derivatives of cholesterol (including estradiol and testosterone). This suggests that impaired cholesterol biosynthesis as well as its further processing lead to the development of various metabolic disorders. It is worth noting that more than half of the total amount of cellular cholesterol is localized inside the plasma membrane, where it is essential for the optimal integrity of the phospholipid bilayer and for proper signal transduction [16]. It has been shown that cholesterol depletion from the plasma membrane leads to improved insulin sensitivity in diseased cells [17], suggesting a role of cellular cholesterol in maintaining the resistance to insulin. Research by Le Lay et al. [18] indicated that there is a possibility to manipulate the cellular insulin response by modifying the cholesterol content of the cells. In healthy cells, insulin stimulates glucose uptake by inducing the translocation of glucose transporters from vesicular structures to the cell surface [19]. Many studies have analyzed the mechanisms by which glucose transporters are translocated to the plasma membrane, but the trafficking pathway of the exocytic vesicles still remains unclear. It was proposed that clathrin-mediated endocytosis and caveolae-mediated endocytosis may be involved in the process [20]. There is evidence indicating that glucose transporters, like glucose transporter 1 (GLUT1) and GLUT4, colocalize with caveolar structures in many cell types [21]. Cholesterol depletion leads not only to the disruption of caveolae but also to the induction of metabolic stress within the cell. A study performed by Barnes et al. [22] showed that methyl-β-cyclodextrin treatment leads to increased activity of the glucose transporters and to their enhanced translocation within the cell. The changes in the distribution of the transporters after the disruption of lipid rafts indicates the role of cholesterol in maintaining glucose homeostasis. Moreover, it was shown that glucose metabolism is strictly dependent on the changes of intracellular cholesterol level [23].

In this study, we decided to use methyl-β-cyclodextrin, a cyclic oligosaccharide which has a capacity to form inclusion complexes with membrane cholesterol, thus increasing its solubility in aqueous solutions [24]. MβCD treatment was described as a possible method for modulating the cholesterol content of cultured cells [25]. The cholesterol management in the cells is correlated with glucose metabolism [26], which suggests that the use of compounds capable of interacting with cellular cholesterol may play a role in the prevention of the development of metabolic-related diseases. It was also suggested that it might be reasonable to use MβCD for IR because of its ability to reduce the level of cholesterol [17]. Moreover, the most common strategy for diabetes treatment is based on the ability of particular drugs, such as metformin and chromium [27], to lower the cholesterol level, which enhances glucose transport mediated by GLUT4 via activation of the 5′AMP-activated protein kinase (AMPK) pathway. We used a low concentration of MβCD (0.5 mM) that affects both caveolae degradation and cholesterol extraction from the plasma membrane [24]. Then, we examined the impact of MβCD on the apoptosis process, proliferation, morphology, and oxidative stress in equine adipose-derived mesenchymal stromal cells to determine the potential use of this drug in cellular therapies. We examined the changes that occur during MβCD treatment in both cells isolated from healthy horses and cells isolated from horses with metabolic syndrome to evaluate the effect of the drug on pathologically changed cells.

## 2. Results

### 2.1. Proliferation of EqASCs

**Figure 1:**
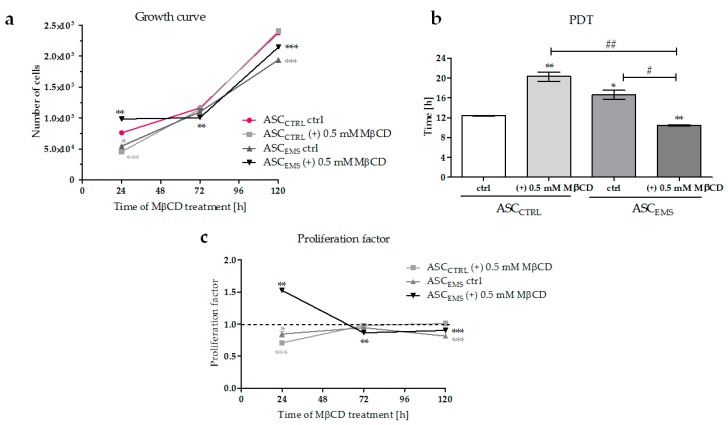
Proliferation of equine ASCs during a five-day culture. The growth of cells (**a**) was determined using a resazurin-based assay (TOX-8). The population doubling time (PDT) was expressed in hours (**b**). The proliferation factor (PF) was calculated in comparison with the control group (**c**). PF values higher than 1 indicated an increased proliferation potential. A hashtag (#) indicates a statistically significant difference between the experimental groups, while an asterisks (*) indicates a statistically significant difference in comparison to the control ASC_CTRL_ group. The results are expressed as mean ± SD; */# *p* value < 0.05, **/## *p* value < 0.01, and *** *p* value < 0.001. ASC_CTRL_—control cells isolated form healthy horses, ASC_EMS_ - cells isolated from individuals with EMS.

The number of proliferating ASCs was determined by measuring the reduction of resazurin to resorufin in the first, third, and fifth day of methyl-β-cyclodextrin treatment. The initial cell seeding density for each group was 20,000 cells per well. After 24 h of MβCD treatment (Figure 1a), the number of proliferating cells decreased in cultures of both ASCs from horses suffering from metabolic syndrome (ASC_EMS_) and cells isolated from healthy horses cultured with an addition of MβCD (ASC_CTRL_ (+) MβCD) compared with ASC_CTRL_ cultures. At the same time, we observed the promotion of cell proliferation in ASC_EMS_ (+) MβCD group, which was manifested by a decrease in the population doubling time (Figure 1b) and by an approximately 50% higher proliferation factor for these cells (Figure 1c). After 72 h of culture in the presence of MβCD, we observed a decrease in the rate of proliferation in ASC_EMS_ (+) MβCD group in comparison with ASC_CTRL_ cells, indicating that the cells had entered a stationary phase of growth. In the last day of experimental treatment, there were no significant differences in the cell number between the control and the experimental groups (Figure 1a,c).

### 2.2. Morphology and Ultrastructure of EqASCs

**Figure 2:**
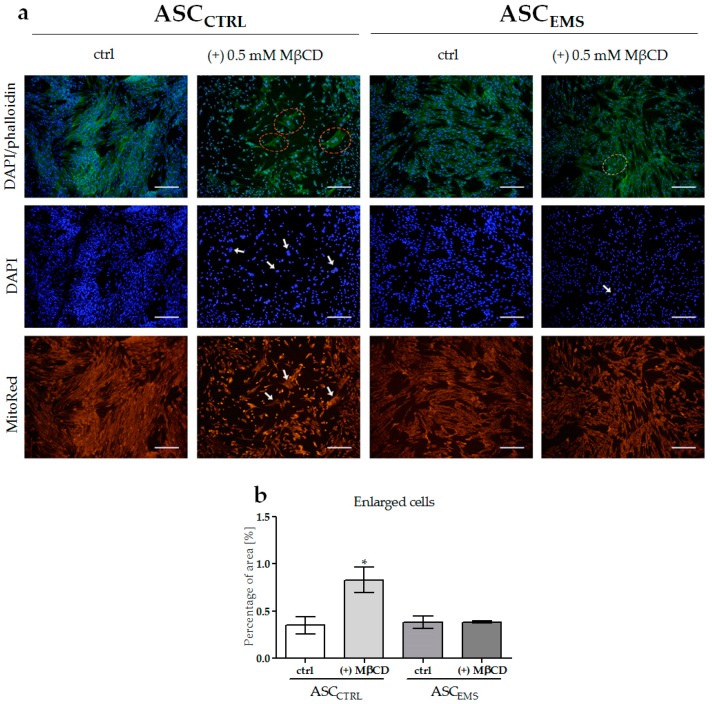
The effect of methyl-β-cyclodextrin on cell morphology. Staining for DAPI, DAPI/phalloidin merged, and MitoRed (**a**). Enlarged cells with oversized nuclei and improper mitochondria distribution are marked on the photographs. The percentage of the enlarged cell area versus the total cell area is presented in (**b**). An asterisks (*) indicates a statistically significant difference in comparison to the control ASC_CTRL_ group. The results expressed as mean ± SD; * *p* value < 0.05. Magnification: DAPI × 100, scale bar: 200 µm; DAPI/phalloidin × 100, scale bar: 200 µm.

On the first day of the experiment, the cells from the control and experimental groups exhibited a proper spindle-shaped morphology. Fluorescent staining (Figure 2a) after 5 days demonstrated that MβCD treatment induced a rapid change in the morphology of control cells, associated with the loss of polarity and mitochondria disturbance (Figure 2a,b). Additionally, we observed a relatively high number of enlarged cells with oversized nuclei in this group, compared with cells isolated from individuals with EMS. MβCD treatment in ASC_EMS_ cells did not cause visible morphological changes in comparison to ASC_EMS_. On the 5th day of the experiment, the cells were characterized by multipolarity and decreased mitochondrial activity. ASC_CTRL_ cultured without MβCD to the last day of the experiment exhibited a typical morphology, with centrally positioned nuclei, well developed actin cytoskeleton, and properly arranged mitochondria.

**Figure 3:**
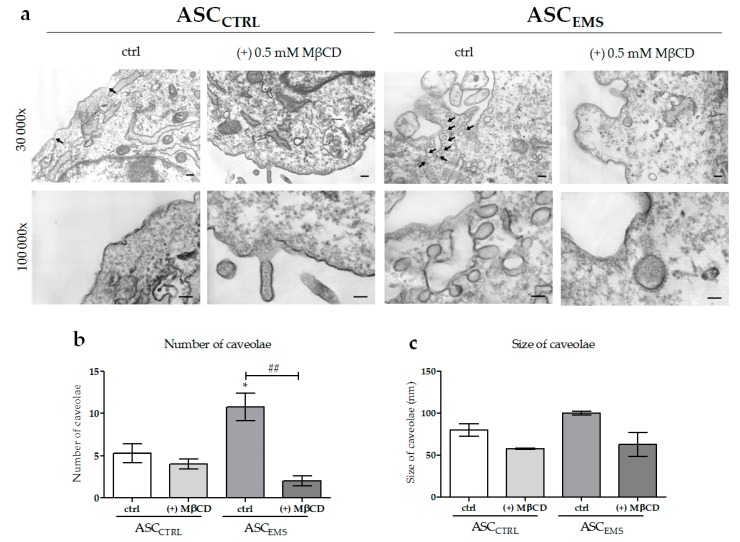
Ultrastructure of EqACSs (**a**). The number (**b**) and size of caveolae (**c**) within the field of view. In TEM images, caveolae are indicated by arrows. Magnification: 30,000× and 100,000×, scale bar: 200 nm for magnification 30,000 and 100 nm for magnification 100,000×. An asterisks (*) indicates a statistically significant difference in comparison to the control ASC_CTRL_ group. The results expressed as mean ± SD; * *p* value < 0.05 and ## *p* value < 0.01.

An utrastructure analysis of the plasma membrane (Figure 3a) was performed by using a transmission electron microscope. We observed significant differences in the amount and size of formed caveolae in ASC_EMS_, in comparison to ASC_CTRL_ cells (Figure 3b,c). ASC_EMS_ exhibited a significantly higher number of endocytic vesicles located inside the cell membrane, which occurred in groups and had much larger structures. The structures occurring within the membrane of healthy cells were less developed and less frequent, when compared with ASC_EMS_. In both cases, we observed a significant reduction of caveolae formation after MβCD treatment.

### 2.3. Oxidative Stress Factors Analysis and the Impact of MβCD Treatment on Endoplasmic Reticulum Stress

**Figure 4:**
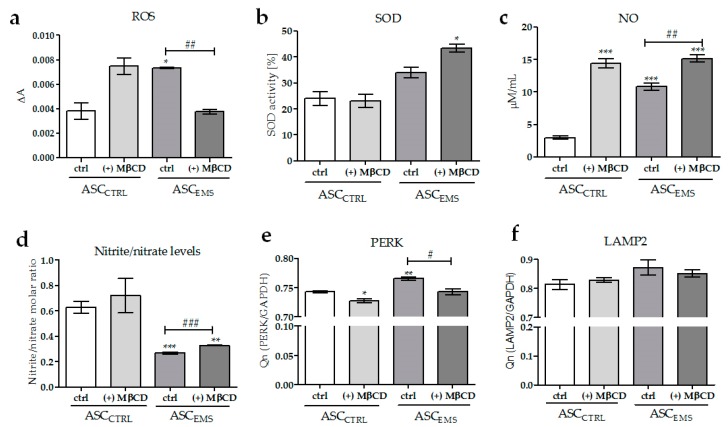
Analysis of the extracellular levels of reactive oxygen species (ROS) (**a**); superoxide dismutase (SOD) activity (**b**); nitric oxide (NO) concentration (**c**) and nitrite-to-nitrate molecular ratio (**d**) in EqASCs. Protein kinase RNA-like endoplasmic reticulum kinase (PERK) and lysosome-associated membrane protein 2 (LAMP2) expression in equine ASCs (**e**,**f**). An asterisks (*) indicates a statistically significant difference in comparison to the control ASC_CTRL_ group. The results expressed as mean ± SD; */# *p* value < 0.05, **/## *p* value < 0.01, and ***/### *p* value < 0.001.

The extracellular reactive oxygen species (ROS) levels were determined by the measurement of the oxidative conversion of non-fluorescent 2′,7′-dichlorodihydrofluorescein into fluorescent 2′,7′-dichlorofluorescein. We showed that ROS production in ASC_EMS_ was reduced as a result of MβCD treatment (Figure 4a). Meanwhile, however, we observed that the ROS level was upregulated in ASC_CTRL_ cultured in the presence of the drug. The comparison of superoxide dismutase (SOD) activity between the groups showed significantly higher level in ASC_EMS_ (+) MβCD compared with the other groups of cells (Figure 4b). The analysis of nitric oxide (NO) synthetase activity revealed that the NO levels significantly increased after MβCD treatment in both ASC_CTRL_ and ASC_EMS_ groups (Figure 4c). Additionally, we observed the upregulation of the nitrite-to-nitrate molar ratio by the action of MβCD in ASC_EMS_ (Figure 4d).

To determine the impact of MβCD on the induction of endoplasmic reticulum stress, we examined *PERK* gene expression. We observed that MβCD treatment caused downregulation of the transcript levels in both ASC_CTRL_ (+) MβCD and ASC_EMS_ (+) MβCD groups (Figure 4e). We also examined the transcript levels of LAMP-2 (Figure 4f). We observed a downward trend in the expression of LAMP2 in ASC_EMS_ (+) MβCD as a result of MβCD treatment. In contrast, we observed an upward trend in ASC_CTRL_ (+) MβCD cultured in the same conditions, but the differences between the groups were statistically insignificant.

### 2.4. The Effects of MβCD on the Apoptotic Process

**Figure 5:**
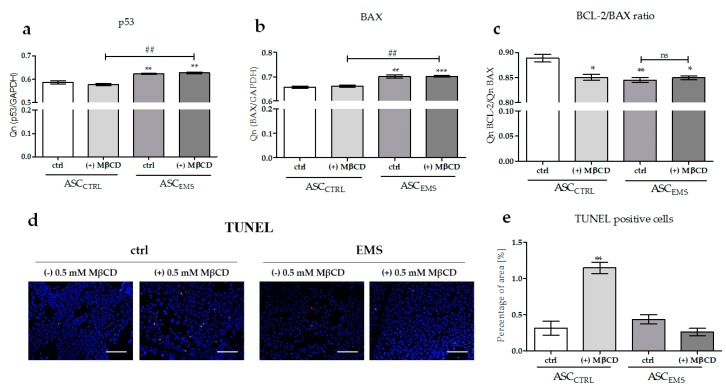
The analysis of the apoptotic process in EqASCs. mRNA levels of p53 (**a**); BAX (**b**); and the BCL-2/BAX ratio (**c**) in control and experimental cultures. The level of expression of the indicated genes was calculated in relation to the housekeeping gene *GAPDH*. An asterisks (*) indicates a statistically significant difference in comparison to the control ASC_CTRL_ group. The results expressed as mean ± SD; * *p* value < 0.05, **/## *p* value < 0.01, and *** *p* value < 0.001. Results of the TUNEL staining (**d**); BrdU-positive cells were stained red. DAPI counterstain was used to determine the total cell number. Percentage of the positive stained area versus the total stained area (**e**). Magnification: ×100, scale bar: 200 µm.

The next stage of this study was to determine the impact of MβCD treatment on the apoptotic process of EqASCs. We examined the changes in the transcript levels of p53, BAX, and BCL-2 (Figure 5a–c) and performed the TUNEL staining (Figure 5d,e) that allows to detect DNA fragmentation by fluorescence microscopy. No statistically significant changes were observed in the mRNA levels assessment by RT-PCR after cyclodextrin application. The comparison of BAX and BCL-2 expression levels showed that MβCD treatment caused a significant reduction of the anti-apoptotic BCL-2 level in ASC_CTRL_, bud did not cause a decline in the BCL-2/BAX ratio in ASC_EMS_. Furthermore, the same regularity could be observed in the number of TUNEL-positive cells. The control cells cultured in the presence of MβCD were characterized by a significantly increased DNA fragmentation when compared with cells cultured in the absence of MβCD. The EqASCs isolated form horses with EMS cultured both in the presence and in the absence of MβCD exhibited a comparable amount of TUNEL-positive cells.

### 2.5. Analysis of the Expression Levels of LAMP-2, GLUT4, and LEP and of the Extracellular Secretion of Adiponectin and Insulin

**Figure 6:**
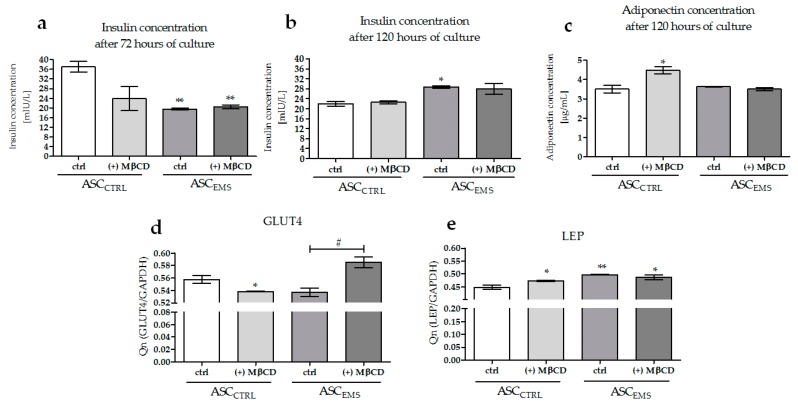
Extracellular levels of insulin (**a**,**b**) and adiponectin (**c**) evaluated by quantitative ELISA. Furthermore, using RT-PCR, the mRNA levels of GLUT4 (**d**) and leptin (LEP, **e**) were assessed. A hashtag (#) indicates a statistically significant difference between the experimental groups, while an asterisks (*) indicates a statistically significant difference in comparison to the control ASC_CTRL_ group. The results expressed as mean ± SD; */# *p* value < 0.05, ** *p* value < 0.01.

ELISA assays were performed to evaluate the levels of insulin (Figure 6a,b) and adiponectin (Figure 6c) in the cell supernatants. We did observe a time-dependent downregulation of the insulin levels in ASC_CTRL_ and ASC_CTRL_ (+) MβCD and an upregulation in ASC_EMS_ and ASC_EMS_ (+) MβCD groups. In both time points, we observed significant differences in the amount of insulin between ASC_CTRL_ and ASC_EMS_, but we did not observe differences caused by MβCD treatment. For comparison, we observed a level-dependent effect of MβCD on the adiponectin amount present in ASC_CTRL_.

RT-PCR detections were performed to evaluate the level of GLU4 (Figure 6d). Compared to ASC_CTRL_, the mRNA expression was downregulated in ASC_CTRL_ (+) MβCD and ASC_EMS_. ASC_EMS_ (+) MβCD exhibited a significantly higher level in relation to ASC_EMS_, and the amount of mRNA was comparable to that observed in ASC_CTRL_. However, LEP mRNA expression (Figure 6e) was significantly upregulated in all groups when compared with ASC_CTRL_, but the comparison of ASC_EMS_ and ASC_EMS_ (+) MβCD showed that MβCD treatment resulted in the downregulation of the mRNA of LEP in cells isolated from horses with EMS, even if the difference was insignificant.

## 3. Discussion

In this study, we examined the effect of methyl-β-cyclodextrin on the biological functions of adipose-derived mesenchymal stromal cells of equine metabolic syndrome-affected horses (ASC_EMS_). We found that this drug had a different biological effect on ASC_EMS_ versus healthy ASCs. The collected data demonstrated that the changes in cellular function due to the application of methyl-β-cyclodextrin were largely dependent on the initial cytophysiological state of the cells. We showed that the application of the methylated form of β-cyclodextrin to healthy ASCs resulted in the distortion of cell morphology and the induction of apoptosis and significantly decreased cell proliferation activity, while cells isolated from individuals with EMS seemed to be more resistant to MβCD treatment. Furthermore, we showed that the application of MβCD significantly increased the proliferative activity of ASC_EMS_, as evidenced by an increase in the population doubling time. A study performed by Portilho et al. suggested that methyl-β-cyclodextrin enhances cell proliferation via activation of the Wnt/β-catenin signaling pathway [28]. The authors proposed that this is likely due to the rapid changes in the cholesterol content in cell membranes affected by MβCD, but the lack of the analysis of the cholesterol levels in our work makes it difficult to transfer this result to the evidence we presented. On other hand, we observed a decrease in the proliferation of ASC_CTRL_ as a result of MβCD application, which is consistent with previous reports that MβCD treatment is associated with changes in the G1 and S phases of the cell cycle. Choi et al. found that cyclodextrin treatment is connected with the downregulation of the expression of cyclin A, which plays a major role in cell growth [29]. Furthermore, we speculate that this may be linked to the observed loss of cell polarity and altered microdomain (e.g., caveolae) organization.

To get insight into the cellular mechanisms underlying metabolic syndrome, we examined the efficiency of caveolae formation. It was previously proposed that caveolae form more readily in the presence of high levels of membrane cholesterol [30]. Furthermore, it is widely acknowledged that the levels of caveolin-1, a major protein of caveolar structures, are increased in the organisms affected by metabolic diseases [31]. The observations of the ultrastructure of the plasma membrane performed in this study showed that ASC_EMS_ were characterized by a significantly greater amount of caveolae located within the membrane, in comparison to the control cells, where caveolae were much less common. It was proposed that membrane microdomains can play an important role in organelle scrambling leading to the formation of autophagosomes [32]. As it was shown by our team’s previous data, ASC_EMS_ are characterized by selective mitophagy over autophagy, which is crucial in the context of their molecular survival [10]. A causal link between mitophagy and MβCD application was previously described in the literature, indicating that the treatment may results in mitophagy-mediated antitumor activity, through the restoration of cell homeostasis by selective degradation of the damaged mitochondria [33] . Furthermore, it was described that β-cyclodextrin, during the course of neurodegenerative diseases such as Niemann–Pick type C disease, which is associated with an abnormal cholesterol accumulation and mitophagy impairment, leads to rescued functionality of the mitochondria [34]. In the both ASC_CTRL_ and ASC_EMS_, we observed decreased caveolae formation as the result of cholesterol depletion, which is consistent with the previous reports [12,35].

Several studies have demonstrated that the abnormalities in membrane fluidity, manifested, inter alia, by impaired caveolae formation, may explain the impaired glucose uptake. It has been proposed that lipid accumulation in the plasma membrane leads to a deregulated structural integrity of the cellular membrane and, thereby, to changes in glucose transport [36]. Rudrappa et al. [37] provided an evidence suggesting that increasing membrane cholesterol content may reduce glucose uptake. In the present study, we demonstrated that the application of cyclodextrin led to increased GLUT4 expression in ASC_EMS_, which is consistent with the findings of Llanos et al. [17] that methyl-β-cyclodextrin treatment influences glucose uptake through GLUT4. Moreover, the findings by Habegger et al. indicated that MβCD treatment may be a useful tool to enhance GLUT4 regulation, which occurs via restoration of F-actin structure and insulin sensitivity [27].

It is worth noting that GLUT4 is a glucose transporter known as an insulin-responsive molecule. The translocation of GLUT4 is required for the proper functioning of cells because of its influence on glucose uptake [35]. Recent studies have shown that abnormalities in the membrane content of GLUT4 lead to impaired insulin sensitivity and, thereby, to insulin resistance or type 2 diabetes [20]. Following this information, we examined the link between extracellular insulin secretion and the expression of the glucose transporter GLUT4. Interestingly, we did not observe a correlation between the rate of extracellular insulin secretion and GLUT4 expression in the diseased cells. This may indicate that MβCD treatment leads to changes in GLUT4 expression levels in an insulin-independent manner. In the last years, it became clear that insulin is not required for the translocation of glucose transporters. Numerous studies conducted on muscles showed that reactive oxygen species (ROS) and nitric oxide (NO) may be involved in the translocation of GLUT4 [38]. In this study, we examined the level of nitrite formed by the spontaneous oxidation of NO and the nitrite-to-nitrate molecular ratio. It was previously shown that the nitrite-to-nitrate molar ratio can be a useful parameter indicating that cholesterol content in the plasma membrane is increased [39]. Here, we observed that the nitrite/nitrate level in EMS-affected horses was significantly decreased when compared with the control culture, which suggests the presence of hypercholesterolemia in this type of cells, but this issue requires additional research. MβCD treatment caused a significant improvement in the content of lipids in EqASC_EMS_. Moreover, we observed a positive regulation of nitrite synthesis in cells isolated from individuals with EMS. This may suggest that MβCD treatment leads to the translocation of glucose transporters through the activation of an NO-dependent signaling pathway. It was shown that nitrite improves glucose tolerance, insulin sensitivity, and dyslipidemia [40], which, together with the results of the study performed by Tanaka et al. [41] that proposed that nitric oxide can stimulate glucose uptake in adipocytes without involving insulin, additionally supports our findings.

Equine metabolic syndrome is also associated with the elevated concentration of adipokines, such as leptin and adiponectin, in peripheral blood [3]. Both hormones play an important role in the development of insulin resistance. We did not observe changes in the amounts of these hormones in ASC_EMS_ after a prolonged treatment with MβCD. This may suggest that the cells generated mechanisms allowing to counterbalance the loss of these hormones. The significant upregulation of both hormones in healthy cells after MβCD treatment can be explained by the impairment of caveolae formation, which additionally supports hormone activity.

We also examined the impact of MβCD on endoplasmic reticulum function, by investigating the molecular changes in the expression of PRKR-like endoplasmic reticulum kinase (PERK). An increased activity of the kinase was observed in β cells as the result of an insufficient amount of glucose in those cells [42], which is comparable to our results. We observed a significant downregulation of PERK expression as the result of MβCD treatment, which might suggest that the incubation of ASC_EMS_ in the presence of the drug led to improved endoplasmic reticulum activity, as well as to increased glucose uptake. Furthermore, we noted an enhanced action of superoxide dismutase (SOD), which, together with the downregulation of ROS activity, suggests that MβCD application was associated with increased antioxidant capacity in ASC_EMS_. Additionally, we investigated the impact of MβCD on membrane-bound organelles, which are linked to insulin resistance [43]. We indicated that methyl-β-cyclodextrin did not affect lysosomal dysfunction, which is consistent with the characteristics of the compound and suggests that cyclodextrin does not affect the functioning of lysosomes. This ability may be a turning point in cellular therapy.

## 4. Materials and Methods

All experimental procedures were approved by the II Local Ethics Committee of Environmental and Life Sciences University (Chelmonskiego 38C, 51-630, Wroclaw, Poland; decision No. 84/2012).

### 4.1. Cell Culture

Equine adipose-derived mesenchymal stromal cells were obtained from the Department of Experimental Biology and Electron Microscope Facility (Wroclaw University of Environmental and Life Sciences); stem cells were collected at passage 3. All the EqASCs populations used in the experiments exhibited a MSC-like antigen profile (high CD90, CD73b, and CD105 expression and lack of CD34 and CD45 hematopoietic markers) and were isolated in accordance with the protocol described by Marycz et al. [3,10].

During the experiments, the cells were cultured in Dulbecco’s Modified Eagle’s Medium (DMEM, Sigma Aldrich, Munich, Germany) containing 4500 mg/L glucose and supplemented with 10% Fetal Bovine Serum (FBS) and 1% antybiotic-antimycotic solution at 37 °C in an incubator with 95% humidity and 5% CO_2_. The media were changed every two days. The cells were passaged after reaching 80% confluence using TrypLE Express (Life Technologies, Warsaw, Poland), according to the instructions provided by the manufacturer.

In order to perform the experiments, the cells were seeded in 24-well plates in the initial number of 2 × 10^4^. The culture medium (0.5 mL/per well) contained 10% FBS, 1% antibiotics solution, and 0.5 mM methyl-β-cyclodextrin (MβCD, Sigma Aldrich, Munich, Germany). The first dose of MβCD-containing medium was added after 24 h, when the cells were attached to the bottom of the plates. The media in the groups cultured with or without MβCD were changed every two days. Cells cultured in the standard medium, prepared as described above, were used as a control.

### 4.2. Cell Proliferation Assay

The cell proliferation rate was determined spectrophotometrically using the resazurin-based in vitro toxicology assay kit (TOX-8, Sigma Aldrich, Munich, Germany) after 24, 72, and 120 h of culture. As described above, the cells were seeded in 24-well plates in the initial concentration of 2 × 104 cells per well. In order to perform the assay, the culture media were discarded and replaced with 350 µL of resazurin dye solution in an amount equal to 10% of the standard culture medium volume, as described in the manufacturer’s protocol. The cells were then incubated with the solution in a humidified incubator for 2 h. After this time, 100 µL of solution collected from each well was transferred to a 96-well plate. The level of bioreduction of the dye was measured using a microplate reader (Spectrostar Nano, BMG Labtech, Ortenberg, Germany). The spectrophotometric detection was evaluated at the wavelength of 600 nm and at the reference wavelength of 690 nm. The test included a blank, containing medium without cells.

To characterize the proliferation rate of EqASCs, we calculated the population doubling time (PDT) and proliferation factor (PF). The computation of the PDT was carried out using an online software [44], designed by V. Roth, and was based on the formula:

$$PDT=\frac{duration*log\left(2\right)}{log\left(FinalConcentration\right)-\;log\left(InitialConcentration\right)}$$

The PDT was calculated in relation to the last day in which a linear increase of the cell number was observed. The PF was calculated in comparison to control the group.

### 4.3. Evaluation of the Cell Morphology and Plasma Membrane Ultrastructure

The evaluation of the cell morphology was performed on the 5th day of culture, using an inverted, epifluorescence microscope (Axio ObserverA1, Zeiss, Jena, Germany). The mitochondria were visualized using a MitoRed dye (Sigma Aldrich, 1 mM, Munich, Germany), diluted in the standard culture media in the ratio of 1:1000. The visualization of actin filaments was performed by washing the cells twice with HBSS (Hanks’ balanced salt solution, Sigma Aldrich, Munich, Germany) followed by fixation with 4% paraformaldehyde for 45 min at RT. After that, the cellular membranes were permeabilized using 0.1% Triton X-100 in HBSS for 20 min. Then, the cells were washed extensively using HBSS for three times and incubated with 350 µL/per well of Atto-488 phalloidin (Sigma Aldrich, Munich, Germany) diluted in HBSS in a ratio of 1:800 (10 nM). After the incubation period, the cells were washed for two times as described above and incubated with 350 µL/per well of 40,6-diamidino-2-phenylindole (DAPI; 1:1000, 5 ug/mL, Sigma Aldrich, Munich, Germany) to visualize the nuclei. The three stainings were performed in the same well and were conducted in the dark.

The ultrastructure of the plasma membranes was observed using a transmission electron microscope (TEM). For this purpose, the cells were fixed with 2.5% glutaraldehyde in 0.1 M cacodylate buffer (pH = 6.8), followed by rinsing with 0.1 M cacodylate buffer (pH = 6.8). Next, the cells were incubated on ice in 2% osmium tetroxide/1.5% potassium ferricyanide in cacodylate buffer. The incubation lasted for 1 h. After incubation, the cells were washed extensively with ultrapure water and incubated overnight in 1% uranyl acetate aqueous solution. Next, the cells were dehydrated in a graded ethanol series (50%, 60%, 70%, 80%, 90%, 2 × 99.8%) and embedded in Agar Low Viscosity Resin (Agar Low Viscosity Resin Kit, Agar, Worcester, UK). The resin blocks were polymerized for 48 h at 60 °C. Ultrathin sections were obtained using an ultramicrotome (Leica EM UC7, Wetzlar, Germany) and collected on copper grids. The samples were observed using a transmission electron microscope (Auriga 60, Zeiss, Jena, Germany) at 120 kV of acceleration voltage.

### 4.4. TUNEL Staining

To evaluate the internucleosomal DNA fragmentation, Abcam’s red fluorescence in situ BrdU DNA fragmentation (TUNEL) assay was performed. The manufacturer’s protocol was optimized for cells cultured in 24-well plates. The following modifications were made: (i) after the 5th day of culture, the cells were fixed using PFA for 40 min at RT and the fixation was followed by membrane permeabilization using 0.1% Triton X-100 for 15 min at RT; (ii) DAPI counterstain was used as the final step of the procedure.

A documentation was made using a Canon PowerShot camera. The images were analyzed using the ImageJ software to indicate statistical differences between the groups. The measurements were made from at least three images. The percentage of the positive staining versus the total stained area was presented in a graph form.

### 4.5. Measurement of the Oxidative Stress Factor

The measurement of reactive oxygen species (ROS), superoxide dismutase (SOD), and nitric oxide (NO) activity was performed using commercially available kits and reagents (H2DCF-DA (Life Technologies, Warsaw, Poland), SOD Assay Kit (Sigma Aldrich, Munich, Germany), and Griess Reagent Kit (Life Technologies, Warsaw, Poland), respectively). The nitrate-to-nitrite molar ratio was measured using a Nitric Oxide Assay Kit (Cayman Chemical, Ann Arbor, MI, USA). All procedures were performed in accordance with the manufacturers' protocols. In order to perform the statistical analyses, each assay was made in triplicate. The culture supernatants used in the tests were collected on the third day of culture.

### 4.6. Analysis of Gene Expression

After five days of culture, the cultures were rinsed twice with HBSS and then were homogenized with 1 mL of TRI Reagent (Sigma Aldrich, Munich, Germany). The total RNA was isolated according to the method described by Chomczynski and Sacchi [45], and the quantity, as well as the quality, of the isolation was determined using a nanospectrometer (WPA Biowave II). Genomic DNA (gDNA) elimination and complementary DNA (cDNA) synthesis were obtained using a PrimeScript™ RT reagent Kit with gDNA Eraser (Perfect Real Time, TaKaRa Clontech, Otsu, Japan), in accordance to the manufacturer’s instructions. The reactions were performed using a T100 Thermo Cycler (Bio-Rad, Berkeley, CA, USA). Each reaction contained 150 ng of total RNA.

Quantitative RT-PCR was carried out by the CFX Connect Real-Time PCR Detection System (BioRad). The PCR reaction mixtures had a total volume of 20 µL and contained 2 µL of cDNA, 500 nM of primers, and SensiFast SYBR & Fluorescein (Bioline, Trento, Italy). The sequences of the primers used in the amplification are listed in Table 1. The relative gene expression (Qn) was calculated in relation to GAPDH housekeeping gene. All reactions were performed in three replicates.

### 4.7. Enzyme-Linked Immunosorbent Assay (ELISA)

To evaluate the concentration of adiponectin and insulin, the supernatants were collected after 72 and 129 h from control and experimental cultures. The assay was performed using a Horse Adiponectin ELISA Kit and Horse Insulin ELISA Kit (MyBioSource, San Diego, CA, USA), in accordance to the manufacturer’s protocol. The samples used in the procedure were undiluted and centrifuged before the analysis. The optical density was read at 450 nm using a spectrophotometer (BMG Labtech, Ortenberg, Germany).

### 4.8. Statistical Analysis

The experiments were performed in triplicate and each measurement was performed in at least duplicate. The unpaired *t*-test analysis or the analysis of variance (ANOVA) with Tukey’s range test (Prism 5.04; GraphPad Software, San Diego, CA, USA) were used to compare the statistical differences among the groups. *p* value <0.05 was considered as a significant difference.

## 5. Conclusions

We have demonstrated that methyl-β-cyclodextrin treatment of mesenchymal stem cells isolated form horses with metabolic syndrome leads to improved glucose transport and endoplasmic reticulum functionality, as well as to a decrease in reactive oxygen species with simultaneous higher antioxidant capacity. We believe that methyl-β-cyclodextrin may promote a wide range of positive actions and thereby might be used effectively in the treatment of metabolic disorders, such as insulin resistance, metabolic syndrome, and diabetes.

## Figures and Tables

**Table 1:** Sequences of the primers used in quantitative reverse transcriptase real-time polymerase chain reaction qRT-PCR.

Gene	Sequence 5′-3′	Amplicon Length (bp)	Accession No.
*GAPDH*	F: GATGCCCCAATGTTTGTGA R: AAGCAGGGATGATGTTCTGG	250	XM_014866500.1
*p53*	F: TACTCCCCTGCCCTCAACAA R: AGGAATCAGGGCCTTGAGGA	252	U37120.1
*BAX*	F: GCCAGCAAATTGGTGCTCAA R: AGCAGTCACTTCCATGGCTC	260	XM_014830923.1
*BCL-2*	F: TTCTTTGAGTTCGGTGGGGT R: GGGCCGTACAGTTCCACAA	164	XM_014843802.1
*LAMP2*	F: GCACCCCTGGGAAGTTCTTA R: ATCCAGCGAACACTCTTGGG	147	XM_014831347.1
*PERK*	F: GTGACTGCAATGGACCAGGA R: TCACGTGCTCACGAGGATATT	283	XM_014852775.1
*LEP*	F: CACACGCAGTCAGTCTCCTC R: CGGAGGTTCTCCAGGTCAT	176	XM_014854289.1
*GLUT4*	F: TGGGCTCTCCGTGGCCATCTT R: GCTGCTGGCTGAGCTGCAGCA	656	XM_014861501.1
